# Induction of Radiodermatitis in Nude Mouse Model Using Gamma Irradiator IBL 637

**DOI:** 10.1159/000524596

**Published:** 2022-04-13

**Authors:** Thoralf Bernhardt, Stephan Kriesen, Katrin Manda, Christin Schlie, Rüdiger Panzer, Guido Hildebrandt, Brigitte Vollmar, Steffen Emmert, Lars Boeckmann

**Affiliations:** ^a^Clinic and Policlinic for Dermatology and Venereology, University Medical Center Rostock, Rostock, Germany; ^b^Department of Radiotherapy and Radiation Oncology, University Medical Center Rostock, Rostock, Germany; ^c^Rudolf-Zenker-Institute for Experimental Surgery, University Medical Center Rostock, Rostock, Germany

**Keywords:** Gamma irradiation, Dosimetry, Dose finding

## Abstract

**Introduction:**

Acute radiodermatitis is a common, though severe, side effect of radiotherapy against cancer that may lead to an interruption or even abortion of the radiotherapy. Mouse models provide an excellent tool to study pathomechanisms of a radiation-induced dermatitis as well as to test and develop novel innovative treatment strategies.

**Objective:**

The aim of this study was to provide an overview of different mouse models and irradiation devices that have been used so far and to describe the process of the induction of a radiation dermatitis in an immune proficient nude mouse model (SKH1-Hr<sup>hr</sup>) using a IBL 637 cesium-137γ-ray machine.

**Methods:**

This process includes the construction of a radiation shielding chamber, restricting the radiation to the right hind leg of the mouse, a dosimetry, and a dose finding study to identify the appropriate irradiation dose to induce a moderate radiation dermatitis.

**Results:**

A radiation shielding chamber was successfully constructed allowing selective irradiation of the right hind leg. A moderate radiodermatitis is induced with irradiation doses in the range of 60–70 Gy under the here described conditions. Symptoms peak about 8 days after irradiation and decrease relatively quickly thereafter. Histological analyses confirmed typical signs of inflammation.

**Conclusion:**

This study describes for the first time a protocol to induce a moderate radiodermatitis in the nude mouse model SKH1-Hr<sup>hr</sup> using a IBL 637 gamma irradiator. This protocol will allow researchers to study novel treatment strategies to alleviate the burden of a radiodermatitis as a side effect of cancer treatment.

## Introduction

Nowadays, more than 50% of all tumor patients receive radiation treatment in the course of their therapy [1]. Radiation-induced dermatitis is a moderate to severe skin reaction in normal tissue that occurs as a side effect of radiotherapy against cancer in the majority of patients [2]. These skin reactions can either be short term during and up to 3 months after radiation treatment or long term, more than 3 months after the end of treatment. It manifests in erythema, epilation, edema, moist desquamation, and ulceration and is usually accompanied by pain and strong pruritus [3]. Radiation-induced dermatitis often leads to an interruption or, in severe cases, even to an abortion of the cancer therapy. A mouse model provides an excellent in vivo system to study the molecular pathomechanisms of radiodermatitis in more detail as well as to test and develop innovative treatment strategies against these painful side effects of radiotherapy. Therefore, a radiodermatitis has to be induced in a defined area of the particular mouse model. In several studies, different devices have been used to induce a radiation dermatitis in mice (Table 1) [4, 5, 6, 7, 8, 9, 10]. The use of X-rays allows the induction of a local radiation dermatitis without harming inner organs of the mice. Hence, not surprisingly in most studies X-ray devices have been used. But for a few studies also the use of gamma irradiation has been reported [6, 9]. These two studies differ in the irradiation dose and in the animals used. Moriyasu et al. [9] irradiated 7-week-old C57BL/6, WBB6F1-W/W^v^, and WBB6F1-+/+ mice with a 40 Gy single dose using a cobalt-60 source, resulting in erythema, edema, epilation, and dry desquamation, observed at 9–15 days post-irradiation. Ertekin et al. [6] irradiated rats with a 30 Gy single dose using also a cobalt-60 irradiation source. Although using a lower dose, Ertekin et al. [6] observed a faster development of the radiation dermatitis. For each device, specific conditions have to be determined in order to induce a radiation dermatitis of the desired intensity in a particular location. In this technical note, we provide an overview of different mouse models and irradiation devices that have been used so far and describe for the first time the use of an IBL 637 gamma irradiator with a cesium radiation source to induce a moderate radiation dermatitis in a nude mouse model (SKH1-Hr^hr^).

## Methods, Procedure, Results

### Construction of Radiation Shielding Chamber

**Fig. 1:**
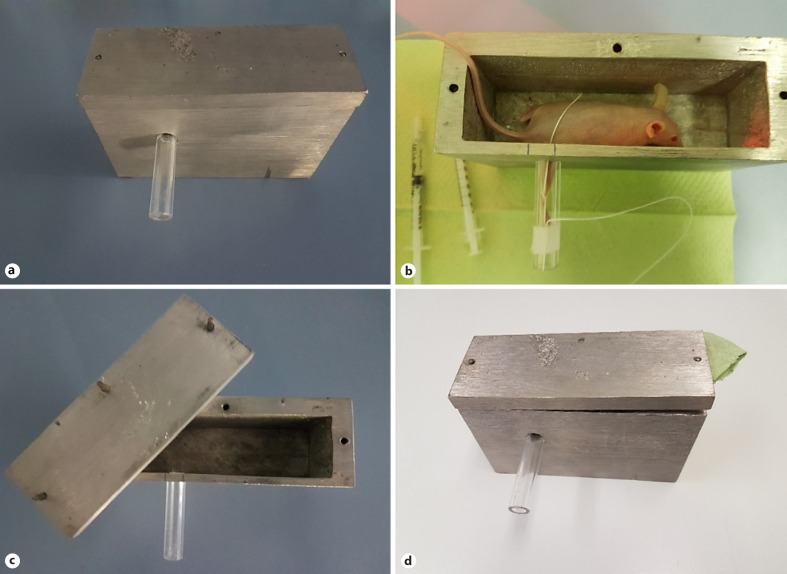
**a** Radiation shielding chamber. **b** Fixated mouse in the radiation shielding chamber. **c** Radiation shielding chamber and lid. **d** Radiation shielding chamber with lifted lid.

In order to achieve a local moderate radiation dermatitis at the right hind leg, a radiation shielding chamber has been constructed which protects the remaining parts of the mouse including inner organs from radiation (shown in Fig. 1a). The chamber and its lid were cast from MCP96 (Rose's metal), which is an alloy of 50% bismuth, 25% lead, and 25% tin. This alloy has a melting point below 100°C and is used in radiation therapy to manufacture individual absorbers. The mold was made from wood and polystyrene. After cooling down, the surfaces were ground in order to obtain a uniform wall thickness.

The 4-cm-thick bottom protects the mouse from the radiation coming from the cesium source (^137^Cs) located below the chamber in the radiation device during the irradiation procedure. The side walls of the chamber only have to protect against scattered radiation and hence do not have to be as thick as the bottom. The front, back, and left walls are 1 cm thick. Only the right side wall is a little thinner (0.5 cm) to ensure that as much as possible of the right hind leg of the mouse can protrude out of the chamber to be fully exposed to the gamma radiation (online suppl. Fig. S1; for all online suppl. material, see www.karger.com/doi/10.1159/000524596). For irradiation, the leg is pulled through a hole in the wall. The hole was drilled into the wall about 3.5 cm from the back of the chamber. Into the hole (diameter: 1.2 cm), a Plexiglas tube (outer diameter: 1.2 cm; inner diameter: 0.8 cm; length: 5.4 cm) was mounted. To pull the leg out of the chamber into the Plexiglas tube, a cord is tied around the leg. By fixing the cord with tape on the outside of the tube, the leg can be fixated in the tube (shown in Fig. 1b).

To protect the mouse also from scattered radiation coming from above, a lid of MCP96 (length: 13.8 cm; width: 5.1 cm; height: 1.1 cm) was constructed and placed on top of the chamber before radiation. Three small pins in the lid that fit into three small holes in the side walls ensure that the lid does not fall off easily (shown in Fig. 1c). If the mice need to be irradiated for more than about 5 min, the lid can be lifted a little bit, for example, by placing some paper between the lid and the chamber (shown in Fig. 1d). This allows air flow into the chamber but still ensures sufficient protection from radiation.

### Radiation Device

**Fig. 2:**
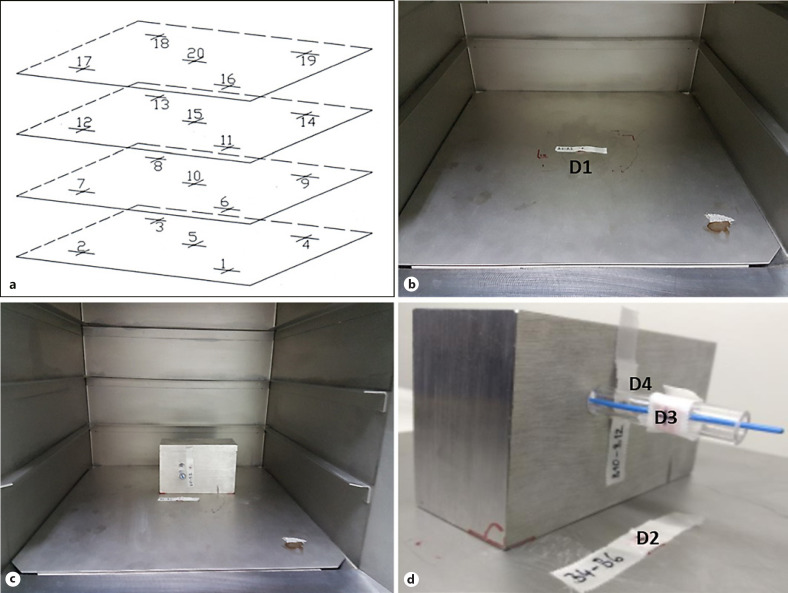
Experimental setup for dosimetry. **a** Objects can be placed at different positions on different planes within the gamma irradiator. **b** A dosimeter (D1) placed at position 5. **c** Radiation shielding chamber inside the gamma irradiator. **d** Radiation shielding chamber inside the gamma irradiator with marked thermoluminescence dosimeters (D2–D4).

Irradiations were performed with a Cesium-137γ-ray machine (Gamma Service Medical GmbH, Leipzig, Germany). Due to the decay of the cesium core, the dose rate of the device decreases over time (exponential decay). Furthermore, the irradiation chamber, which is located above the cesium source, has 4 different levels of exposure (Fig. 2a, c), and at each level, the samples can be placed at different positions (labelled 1–20 in Fig. 2a). Considering that the dose rate also decreases as a function of the distance to the cesium source, the actual dose rate was calculated before use to determine the required exposure time to achieve the desired dose at a specific position in the radiation chamber on a given day. For example, on June 17, 2019, a dose rate of 2.37 Gy was calculated. This means 253 s was required to achieve 10 Gy at position 5 (shown in Fig. 2a). On March 15, 2021, the dose rate was 2.27 Gy/min (264 s/10 Gy at position 5). For the actual irradiation process, the desired time is programed into the irradiation device. The cesium core (irradiation source) is then automatically moved from a shielded position inside the Cesium-137γ-ray machine to a position underneath the irradiation chamber. As soon as the programed time is up, the cesium core is relocated to the original shielded position.

### Dosimetry

In order to test if the constructed chamber sufficiently protects the mouse from irradiation, three independent dosimetries using multiple thermoluminescence dosimeters at different positions in and outside of the radiation shielding chamber were performed. The first two dosimetries have been done with three single thermoluminescence dosimeters to measure the actual radiation at and in the radiation shielding chamber. For the third dosimetry, a hose dosimeter containing 12 thermoluminescence dosimeters (H1-H12) and four separate thermoluminescence dosimeters were used.

First, only one dosimeter (D1 in Fig. 2b) was placed at the center of the irradiation chamber (position 5; see Fig. 2a) and irradiated without the shielding chamber being placed in the irradiation device. Position 5 is closest to the radiation source, and hence, the highest radiation intensity is expected at this position. Irradiation time was calculated to achieve a dose of 8 Gy at position 5. Subsequently, the chamber was placed in the gamma irradiator IBL 637 so that the Plexiglas tube was positioned right at the center at position 5 (shown in Fig. 2c). The hose dosimeter was placed inside the shielding chamber and inside the Plexiglas tube (shown in Fig. 2d). The other dosimeters were placed at position 5 (D2 in Fig. 2d), at the side of the Plexiglas tube (D3 in Fig. 2d), and at the shielding chamber next to the Plexiglas tube (D4 in Fig. 2d). Now, irradiation was turned on again for the same time.

The dosimetry revealed that almost no radiation (0.11 Gy ± 0.06 Gy) is coming through the radiation shielding chamber, whereas outside of the chamber (Table 2, D1–D4 measurements, doses from 4.56 to 8.29 Gy) and inside the Plexiglas tube (Table 3, H1–H9 measurements, dose = 5.4 Gy ± 0.2 Gy) significant radiation was measured. Inside the chamber, close to the Plexiglas tube 0.3 Gy was detected (H12 in Table 3). The radiation measured inside the Plexiglas tube was not as high as the expected value for position 5 (8 Gy). This deviation can be explained by the higher position of the Plexiglas tube compared to position 5 (see Fig. 2b, d). Position 5 is located 25 cm above the radiation source. Because the Plexiglas tube is positioned about 4 cm above position 5 (i.e., at 29 cm above the radiation source), the expected radiation for this position according to the inverse square law is 8 Gy × (25/29)^2^ = 5.9 Gy. Hence, the actually measured value of 5.54 Gy outside the tube (D3 in Table 2) and 5.4 Gy inside the tube (H1–H9, Table 3) is close to the expected value. Considering that the accuracy of the thermoluminescence dosimeters is ±10%, the measured value lies within the expected range of 5.31–6.49 Gy.

Since the right hind leg of the mouse will be positioned in the Plexiglas tube starting close to the wall of shielding chamber and extending inside the tube over few centimeters, we focused on values measured at H9 and H10 (4.05–5.25 leading to a mean value of 4.65 Gy). Therefore, irradiation times for subsequent experiments are calculated based on the here determined difference between position 5 (8 Gy) and the Plexiglas tube (H9 and H10, 4.65 Gy). For example, to achieve an irradiation dose of 8 Gy the calculated time for position 5 was corrected by the factor 1.7204 (8 Gy/4.65 Gy). This extended treatment time should give rise to a dose of 7 Gy inside the Plexiglas close to the wall of the shielding chamber and 9 Gy a bit further away.

### Dose Finding

In order to determine the optimal radiation dose to induce a moderate radiation dermatitis which comes with erythema, hair loss, and moist desquamation (score 2.5 according to Holler et al. [11]; Table 4), different doses have been tested. Therefore, 16 mice were divided into four groups consisting of 4 animals each. These groups were irradiated in accordance with the typical clinical treatment schedule for 5 consecutive days with a total dose of 25, 30, 35, or 40 Gy, respectively. A single irradiation treatment in contrast to a consecutive treatment schedule would require relative long treatment times and, hence, would require the animals to stay narcotized for a long time in the irradiation shielding chamber. As there is no temperature control to keep the mice warm in the irradiation shielding chamber and because oxygen levels may drop over time, a prolonged time in the irradiation shielding chamber should be avoided. Before irradiation, the mice were anesthetized with ketamine/xylazine (98 mg/kg ketamine and 6.5 mg/kg xylazine) and then placed in the radiation shielding chamber where the right hind leg was inserted in the Plexiglas tube and fixed with a cord as described above. At last, the shielding chamber was closed with a lid and placed in the gamma irradiator at the position with the highest irradiation intensity (position 5). After irradiation, the mice were scored every day to assess their health condition and the development of a radiation dermatitis.

**Fig. 3:**
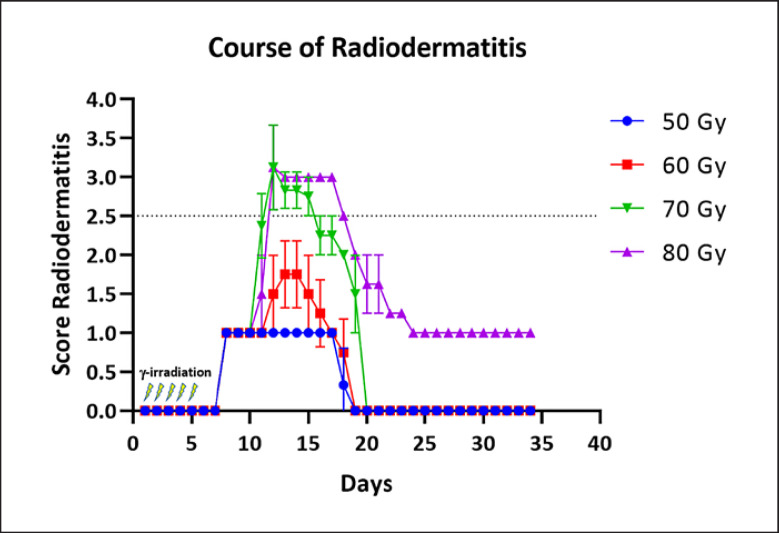
Course of radiation dermatitis. A total of 16 animals have been assessed and were scored using the scoring system represented in Table 4. Mean score and standard deviation (error bars) of four animals per group are shown.

**Fig. 4:**
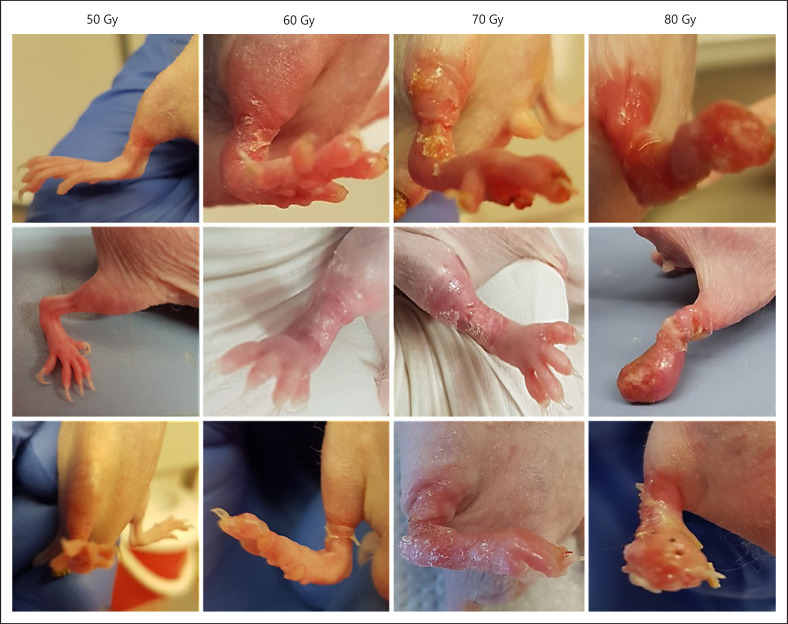
Examples of radiation dermatitis induced with increasing radiation doses. From left to right: 50 Gy, 60 Gy, 70 Gy, 80 Gy. With the occurrence of the first symptoms, mice received an analgesic (Novaminsulfon, 200 mg/kg body weight) via drinking water until the end of the study. It is not expected that this pain reliever has any significant influence on the course or characteristics of the radiodermatitis.

Because no symptoms of a radiation dermatitis besides hair loss (although these mice are nude, they still have some hair) and loss of nails were observed after at least 6 weeks, a second dose finding trial with a new set of 16 animals was performed. This time, total irradiation doses of 50, 60, 70, and 80 Gy were tested by extending the irradiation time. The actually used irradiation time was, for example, 435 s per day (10 Gy) to achieve a total dose of 50 Gy after 5 days. However, this time has to be recalculated before each experiment considering the decay of the cesium source. First symptoms, like hair and nail loss, have been observed already 2 days after the end of irradiation, and in all groups, the radiation dermatitis reached its climax after about 8 days after the end of irradiation (shown in Fig. 3). The 50 Gy group showed slight erythema and dry desquamation (score 1), and the 60 Gy group showed moderate to distinct erythema and spotted moist desquamations (score 2). Mice in the 70 Gy group showed moist desquamations in larger areas (score 2.5), and beyond that, some animals already showed a confluent moist desquamation (score 3). The 80 Gy group showed ulceration of all skin layers as well as confluent desquamations (score 3–4, shown in Fig. 4). These symptoms completely disappeared about 15 days after the end of irradiation for the groups below 80 Gy. Animals in 80 Gy group still showed some symptoms (score 1) until the end of the observational period (day 34).

Based on these results, a moderate radiation dermatitis is induced with irradiation doses in the range of 60–70 Gy under the here described conditions using a IBL 637 Cesium-137γ-ray machine (shown in Fig. 4). A list of the main materials required to set up the here described model to study radiation-induced dermatitis as well as to test and develop novel innovative treatment strategies for this condition is provided in Table 5.

**Fig. 5:**
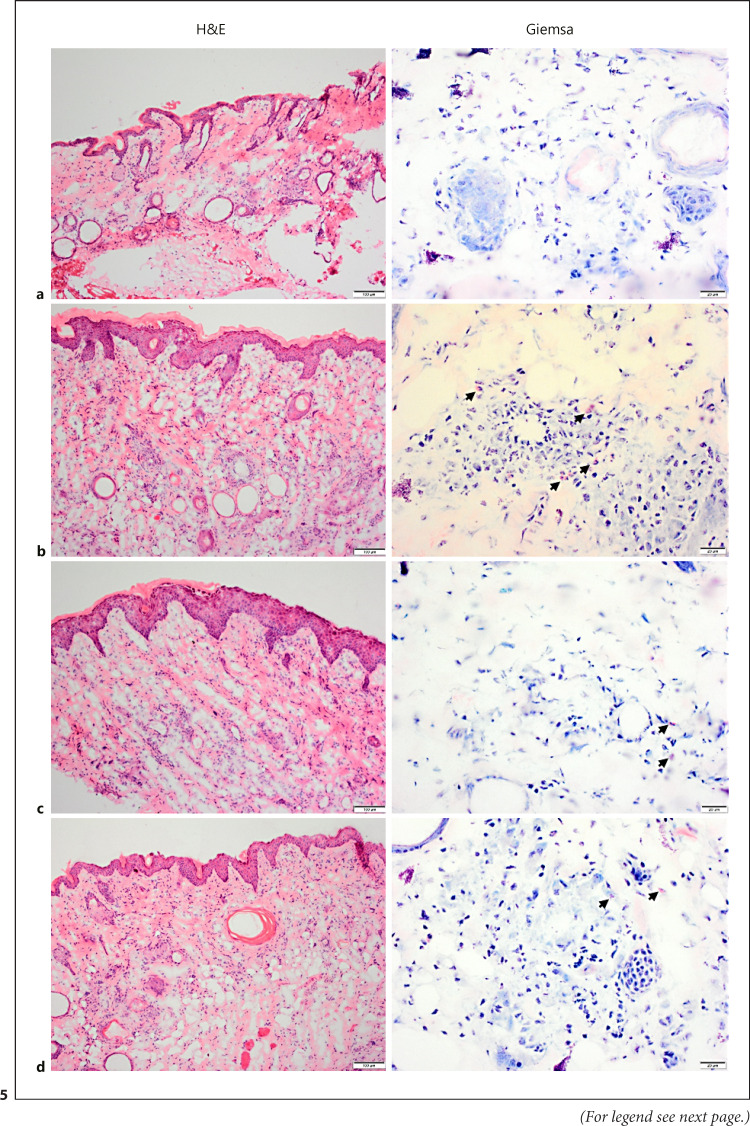
Representative images of histomorphological analyses. Cryosections stained with H&E (left) or Giemsa (right). **a** Cryosections from tissues of a healthy control mouse. **b** Cryosections from tissues of a mouse at the peak of a moderate radiodermatitis (score 2.5). **c** Cryosections from tissues of a mouse where the symptoms have already improved (score 1.25). **d** Cryosections from tissues of a mouse where the symptoms have disappeared (macroscopically healed, score 0).

To further validate the induction of a radiodermatitis, we performed histological analysis. Therefore, cryosections from tissues of mice at the peak of a moderate radiodermatitis (score 2.5) as well as from mice where the symptoms have already improved (score 1.25) or disappeared (macroscopically healed, score 0) were assessed (Fig. 5). Tissues from the healthy left hind leg as well as from a nonirradiated mouse served as controls. Hematoxylin and eosin (H&E) staining of tissue sections revealed a thickening of the epidermis and hyperkeratosis in irradiated tissues. The *stratum corneum* and the *stratum granulosum* are notably thicker compared to untreated tissues. These alterations of the epidermis are still clearly visible even after complete macroscopic healing (Fig. 5d). H&E staining as well as additional Giemsa staining also revealed typical signs of an inflammation.

These cell infiltrates appeared closer to the epidermis at later stages of the radiodermatitis compared to the time point at the peak of the symptoms. Eosinophil granulocytes have also been detected at all three time points, and periodic acid-Schiff reaction revealed some neutrophil granulocytes, whereas cell infiltrates were less obvious after periodic acid-Schiff reaction. Histological analyses of the healthy left legs of the gamma-irradiated mice did not show any alterations compared to the analyses of tissues of the nonirradiated mouse (data not shown). Taken together, histological assessment showed typical signs of inflammation as expected and, hence, confirms the successful induction of a moderate radiation dermatitis in this nude mouse model.

## Discussion

Acute radiation-induced dermatitis is a common and severe side effect of radiation therapy. A substantial number of all cancer patients receiving radiotherapy develop a radiation dermatitis, causing an interruption or even abortion of the radiotherapy and a decrease of life quality. Currently, little is known about the pathomechanisms of a radiation dermatitis and no effective therapies exist. Therefore, novel therapy options that allow an uninterrupted treatment of cancer patients are needed. In order to provide a tool to study pathomechanisms and novel treatment strategies, we here described the induction of a radiodermatitis in a nude mouse model using an IBL 637 Cesium-137γ-ray machine. To the best of our knowledge, there are only a few other examples in the literature describing the induction of a radiation-induced dermatitis in a mouse model by gamma irradiation [6, 9]. Moriyasu et al. [9] treated a hind leg of C57BL/6 mice using a cobalt-60 gamma irradiation source and observed first symptoms about 10 days after a single dose with 40 Gy (dose rate 1.94 Gy/min). The score of the radiodermatitis increased until about day 25 and remained at this level for the rest of the study period until day 60. Ertekin et al. [6] also used a cobalt-60 gamma irradiation source and treated the right hind leg of Sprague-Dawley rats with a single 30 Gy dose. These animals developed first symptoms already 3 days after irradiation, and the score increased steadily over time until day 36 (last day of the study). In our study, however, the highest score was reached about 8 days after the last irradiation and decreased to almost complete healing within the following 8 days. Besides the gamma irradiation source (cesium-137 vs. cobalt-60), our study differed in multiple ways from these studies including the mouse model, total irradiation dose, dose rate, and treatment scheme (fractionated treatment vs. single dose). The age of the mice may also be relevant; Moriyasu et al. [9], for example, used 7-week-old mice, whereas in our study animals with an age of 10–12 weeks were used. All of these parameters may contribute to the difference in the course of the induced radiodermatitis.

The course of the disease also varies a lot in other studies using X-rays to induce the radiation dermatitis. While some authors describe first symptoms already 6 days after irradiation, others observed symptoms only as late as 5 weeks after treatment (Table 1). In the only other studies that also used SKH1-Hr^hr^ mice and fractionated treatment (total dose 48 Gy, 12 Gy/day, dose rate 3 Gy/min), first symptoms were observed about 24 days after treatment [5]. However, besides using X-rays instead of gamma irradiation from a cesium-137 source, the radiodermatitis has also been induced in a different region of the body (posterior dorsal). Overall relatively high doses of gamma irradiation were required to induce a moderate radiation dermatitis in this study (60–70 Gy) compared to other studies (30–60 Gy; see Table 1).

Taken together, many parameters such as radiation source, animal model, treatment/fractionation scheme, radiation dose, and body part may influence the induction of a radiodermatitis. Therefore, a dose finding study has to be conducted for every new experimental setup. In this study, we describe for the first time a protocol to induce a moderate radiodermatitis in the nude mouse model SKH1-Hr^hr^ using a IBL 637 gamma irradiator. This protocol will allow researchers to study the pathomechanisms of radiation-induced dermatitis in more detail as well as to assess novel treatment strategies to alleviate the burden of a radiodermatitis as a side effect of cancer treatment.

## Statement of Ethics

The use of animals in this study has been approved by local regulatory authorities (Landesamt für Landwirtschaft, Lebensmittelsicherheit und Fischerei Mecklenburg-Vorpommern; reference number: 7221.3-1-047/18).

## Conflict of Interest Statement

The authors have no conflicts of interest to declare.

## Funding Sources

This work was supported by the Damp Foundation (2017-05). The funder had no role in the study design, collection, analysis, or interpretation of the data, writing of the manuscript, or the decision to submit the paper for publication.

## Author Contributions

The study was designed by Steffen Emmert and Lars Boeckmann. Thoralf Bernhardt, Stephan Kriesen, Christin Schlie, and Lars Boeckmann performed the study. Thoralf Bernhardt, Katrin Manda, Rüdiger Panzer, Steffen Emmert, and Lars Boeckmann were responsible for the data analysis and interpretation. Administrative, technical, and material support was provided by Katrin Manda, Brigitte Vollmar, Guido Hildebrandt, Steffen Emmert, and Lars Boeckmann. Thoralf Bernhardt provided a first manuscript draft; Lars Boeckmann and Katrin Manda reviewed and edited the script. All authors have participated in the critical revision of the manuscript with regard to important intellectual content.

## Data Availability Statement

All data generated or analyzed during this study are included in this article. Further inquiries can be directed to the corresponding author.

## Supplementary Material

Supplementary data

Supplementary data

## Figures and Tables

**Table 1:** Animal models, radiation devices, and conditions described in the literature to induce a radiation dermatitis

Reference	Animal	Radiation source	Radiation dose (dose rate)	Radiation fractions	Region	First symptoms
Cho and Kim [5] Early diagnosis of radiodermatitis using lactate dehydrogenase isozymes in hairless mice	SKH1-Hr^hr^ mice	X-ray Mevatron 6700, Siemens, Germany	48 Gy (3 Gy/min)	4 days in a row 12 Gy/day	Posterior dorsal region	24 days after irradiation

Chi et al. [4] Vitamin E deficiency did not exacerbate partial skin reactions in mice locally irradiated with X-rays	Hairless Hos:HR-1 mice	X-ray generator (200 kV and 20 mA, Pantak HF-320, Shimadzu, Kyoto, Japan)	50 Gy (1.27 Gy/min)	Single dose	Right hind leg	6 days after irradiation

Murakami et al. [10] The effect of azelastine on acute radiation dermatitis in mice models	C3H/He mice	X-ray 230 kV	20, 40, 60 Gy (0.62 Gy/min)	Single dose	Right hind leg	10 days after irradiation

Fares [7] An innovative complex of benzene-poly-carboxylic acid and molybdenum, for prevention and treatment of radiation dermatitis	BALB/cfC3H mice	Medical linear accelerator (Elekta AB, Stockholm, Sweden)	30 Gy (2.5 Gy/min)	5 times	Right hind leg	5 weeks

Maeng et al. [8] Altered immune cell proportions in the radiodermatitis Induced hairless mice-1 (HR-1)	HR-1 mice	X-ray	40 Gy (4 Gy/min)	4 consecutive days 10 Gy/day	Posterior dorsal region	10 days after first irradiation

Moriyasu et al. [9] Involvement of histamine released from mast cells in acute radiation dermatitis in mice	C57BL/6 mice	Gamma irradiator (cobalt-60)	40 Gy (1.94 Gy/min)	Single dose	Hind leg	10 days

Ertekin et al. [6] The effect of zinc sulfate in the prevention of radiation-induced dermatitis	Sprague-Dawley rats	Gamma irradiator (cobalt-60)	30 Gy (0.68 Gy/min)	Single dose	Right hind leg	3 days

This study	SKH1-Hr^hr^ mice	Gamma irradiator IBL637 (cesium-137)	50–80 Gy (1.38 Gy/min)	5 consecutive days 10–16 Gy/day	Right hind leg	2 days after irradiation time

**Table 2:** Detected radiation doses with thermoluminescence dosimeters D1-D4

Dosimeter	Position	Radiation dose, Gy (mean of 3 measurements)
D1	Position 5 (without shielding chamber)	8.29±0.07

D2	Position 5 (with shielding chamber)	7.91±0.29

D3	Laterally at Plexiglas tube	5.54±0.21

D4	At shielding chamber next to Plexiglas tube	4.56±0.04

**Table 3:** Detected radiation doses with thermoluminescence dosimeters within the hose

**Figure:** 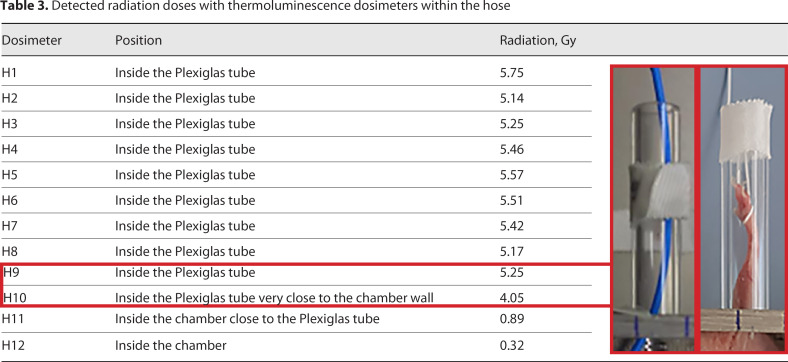 **Figure:** 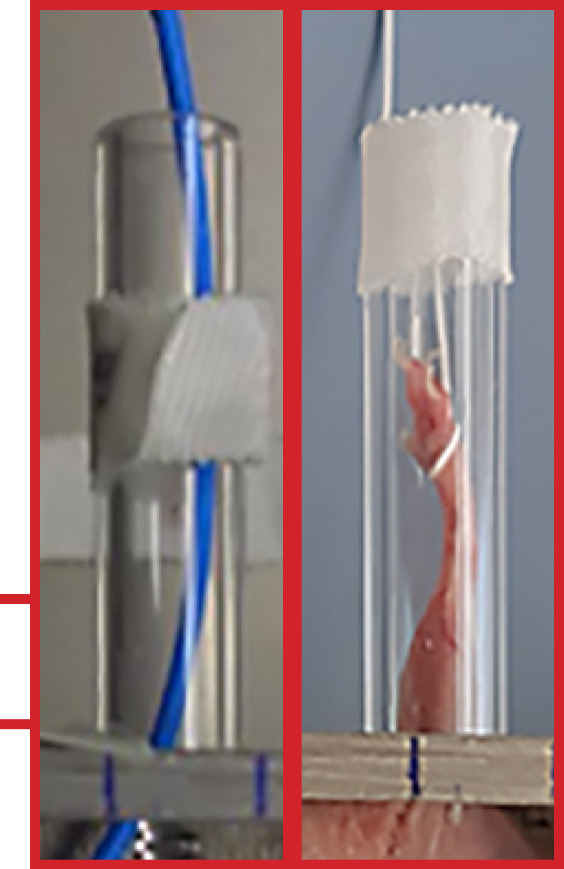

**Table 4:** Radiation dermatitis score modified after Holler et al. [11]

Score	Symptoms
0	No visible skin irritation
1	Slight erythema or dry desquamation
1.25	Distinct erythema
2	Moderate erythema or stained moist desquamation (25% of irradiated skin)
2.5	Moist desquamation in a bigger area (50% of irradiated skin)
3	Confluent moist desquamation
4	Skin necrosis or ulceration of all skin layers

**Table 5:** Main materials

Material
Mice (SKH1-Hr^hr^)
Gamma irradiator (IBL 637)
Thermoluminescence dosimeters
Hose dosimeter
Radiation shielding chamber (cast from MCP96)
Anesthetics (ketamine/xylazine, Novaminsulfon)
Syringe/cannulae
