# Supplementary material for: Induction of Radiodermatitis in Nude Mouse Model Using Gamma Irradiator IBL 637
Source: Skin Pharmacol Physiol. 2022 Apr 13;35(4):224–34. doi: 10.1159/000524596 (PMC9254314; doi:10.1159/000524596)
Supplement: Supplementary file 1 — Supplementary data [file spp-0035-0224-s01.docx]

Figure Legends Online Supplementary Material

Fig. S1. Technical drawing of the radiation shielding chamber.
