# Supplementary figures and images for: Induction of Radiodermatitis in Nude Mouse Model Using Gamma Irradiator IBL 637
Source: Skin Pharmacol Physiol. 2022 Apr 13;35(4):224–34. doi: 10.1159/000524596 (PMC9254314; doi:10.1159/000524596)

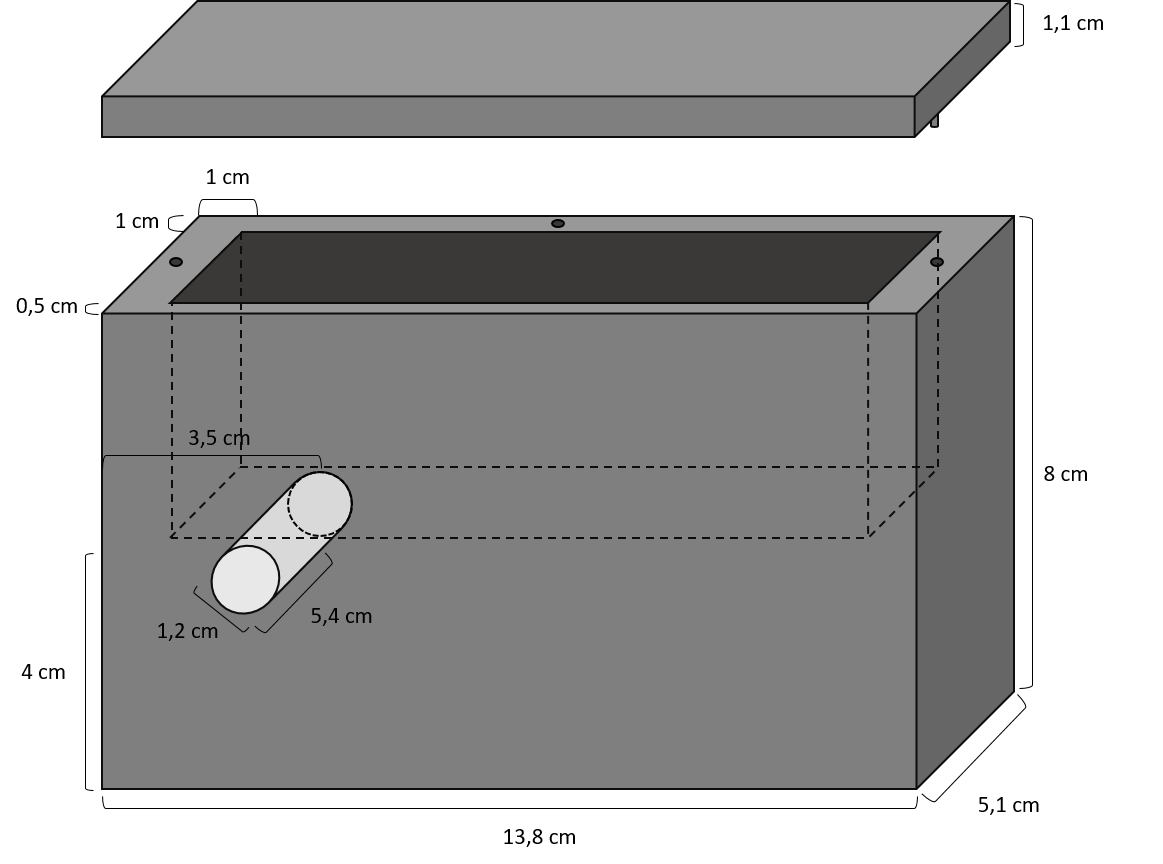

Supplement: Supplementary file 2 — Supplementary data [file spp-0035-0224-s02.png]
